# Immunologic shifts occurring during cow milk (CM) oral immunotheray (OIT)

**DOI:** 10.1186/1939-4551-8-S1-A196

**Published:** 2015-04-08

**Authors:** Alfredo Alves Neto, Conrado Martins, Priscila Aboud Pimenta, José Luiz De Magalhães Rios, Fabio Kuschnir, Marilucia Alves Da Venda, João Bosco Magalhães Rios

**Affiliations:** 1 Policlinica Geral Do Rio De Janeiro, Brazil; 2 Faculdade De Medicina De Petrópolis – Fase, Brazil; 3 Rio De Janeiro State University, Brazil

## Background

The Paradigm of cow’s milk allergy (CMA) management has shifted in the last years, with the introduction of the Oral Induction Tolerance (OIT) protocols to CMA. Patients with anaphylaxis have persistent and high levels of specific IgE to milk proteins, mainly casein. The purpose of this research was follow the evolution of these parameters during the different phases of CM’s OIT.

## Methods

Series of cases involving 15 children over 4 years and adolescents who still had anaphylaxis to cow's milk. Specific IgE levels were evaluated in three steps of OIT: at baseline, pre-treatment session (step 1); When the patient reached the concentration 1:1 (step 2); and when reaching the final volume to 150 ml of milk a day (step 3). The differences between the levels of specific IgE were analyzed by Student's t test. The adopted level of significance was <0.05

## Results

The age mean of the sample was 8.73 years (min: 4, Max: 19), 9 females. At step 1, the mean for specific IgE levels for milk; casein , α-lacto albumin and B-lacto albumin were respectively : 43,96 KU/L (Min:9,0 KU/l;Max: 100,0 KU/L); 31,35 KU/L (Min:7,0 KU/l;Max: 69,3 KU/L); 18,663 KU/L (Min:1,0 KU/l;Max: 45,5 KU/L) and 10,247 KU/L (Min:2,3 KU/l ; Max: 29,7 KU/L). At step 3, these values were respectively of 19,48 KU/L (Min:2,70 KU/l ;Max: 46,20 KU/L); 17,29 KU/L (Min:1,80 KU/l ; Max: 45,5 KU/L); 2,046 KU/L (Min:0,0 KU/l and Max: 29,5 KU/L) and 4,91 KU/L (Min:1,0 KU/l;Max: 17,0 KU/L). The compare of the mean of specific IgE levels between the steps 1 and 3 reached statistical significance for all antigens: milk (*p*<*0*,*001*); casein ( p=0,003); α-lacto albumin (*p*=*0*,*002*) and B-lacto albumin (*p*=*0*,*005*).

## Conclusions

OIT to anaphylactic CMA reduces the specific IgE levels for milk proteins in parallel to developing of clinical tolerance to high volumes of milk ingestion.

